# Distinct Failure Patterns in Hypopharyngeal Cancer Patients Receiving Surgery-Based Versus Radiation-Based Treatment

**DOI:** 10.1245/s10434-022-12744-1

**Published:** 2022-11-06

**Authors:** Yu-Hsuan Lin, Jenn-Ren Hsiao, Yuan-Hua Wu, Jeffrey S. Chang, Chun-Yen Ou, Wei-Ting Lee, Cheng-Chih Huang, Chan-Chi Chang, Yu-Hsuan Lai, Sen-Tien Tsai, Wei-Ting Hsueh, Chia-Jui Yen, Chen-Lin Lin, Yu-Shan Chen, Shih-Sheng Jiang, Yu-Chu Su, Shang-Yin Wu

**Affiliations:** 1 Institute of Clinical Medicine, College of Medicine, National Cheng Kung University, Tainan, 70456 Taiwan; 2 Department of Otolaryngology, Head and Neck Surgery, Kaohsiung Veterans General Hospital, Kaohsiung, Taiwan; 3 Department of Oncology, National Cheng Kung University Hospital, College of Medicine, National Cheng Kung University, Tainan, 70456 Taiwan; 4 Department of Radiation Oncology, National Cheng Kung University Hospital, College of Medicine, National Cheng Kung University, Tainan, 70456 Taiwan; 5 National Institute of Cancer Research, National Health Research Institutes, Tainan, 70456 Taiwan; 6 Department of Otolaryngology, National Cheng Kung University Hospital, College of Medicine, National Cheng Kung University, Tainan, 70456 Taiwan; 7 Department of Nursing, National Cheng Kung University Hospital, College of Medicine, National Cheng Kung University, Tainan, 70456 Taiwan; 8 Clinical Medicine Research Center, National Cheng Kung University Hospital, College of Medicine, National Cheng Kung University, 70456 Tainan, Taiwan

## Abstract

**Background:**

To cure advanced hypopharyngeal squamous cell carcinoma (HPSCC), primary operation followed by adjuvant (chemo-)radiotherapy (OP-CRT) or definitive chemoradiation (CCRT) are the two primary options. This study aimed to compare the failure patterns and long-term survival outcomes of HPSCC patients treated with these two strategies.

**Patients and Methods:**

From 2007 to 2015, 198 pathologically confirmed HPSCC patients receiving either OP-CRT or CCRT were retrospectively reviewed. Failure patterns and survival outcomes stratified by the 7th American Joint Committee on Cancer staging system and treatment modalities were compared.

**Results:**

One hundred and eighty-nine patients (95.4%) were stage III/IV and 62 patients (31.3%) received OP-CRT. Median follow-up duration was 4.9 years. Compared with CCRT, OP-CRT provided better 3-year local relapse-free survival for T3 (93 vs 48%, *p* < 0.0001), T4a (88 vs 37%, *p* = 0.0005) and better 3-year regional relapse-free survival for N2b+2c (93 vs 60%, *p* < 0.0001). Of note, for stage IVA subjects, OP-CRT provided better 3-year loco-regional relapse-free survival (85 vs 37%, *p* < 0.0001), marginal poor 3-year distant metastasis-free survival (62 vs 79%, *p* = 0.06), but comparable 3-year OS (52 vs 44%, *p* = 0.37) and 5-year OS (44 vs 31%, *p* = 0.15) compared with CCRT.

**Conclusions:**

For patients with advanced HPSCC, although OP-CRT and CCRT provided similar overall survival, failure patterns were distinct. OP-CRT provided better loco-regional control but was more likely to encounter distant metastases than CCRT. The detailed analysis of failure patterns will pave the way to improve this devastating disease.

**Supplementary Information:**

The online version contains supplementary material available at 10.1245/s10434-022-12744-1.

Head and neck cancer (HNC), including cancers arising from the lip and oral cavity, oropharynx, hypopharynx, and larynx, ranked the 7th among the most common cancers, with over 740,000 new cases annually worldwide.^1^ While comprising only about 11.3% (84,254 cases) of HNC,^1^ hypopharyngeal squamous cell carcinoma (HPSCC) has the poorest prognosis compared with cancers arising from other subsites of the head and neck region.^2–5^ The poor outcome of HPSCC mainly comes from the difficulty in early diagnosis, the high propensity of lymph node and distant metastasis at presentation, frequently associated comorbidities, and the occurrence of second primary tumors.^5,6^ Even though some population-based studies demonstrated improving treatment outcomes in recent decades,^3,4^ the prognosis of locally advanced HPSCC remains dismal, with 5-year OS ranging from 15 to 45%.^7^

Primary curative operation followed by adjuvant (chemo-)radiotherapy (OP-CRT) or definitive chemoradiation (CCRT) is the mainstay treatment of HPSCC. Because radical surgery, especially the procedures performed in patients with advanced T-stage diseases, may cause significant swallowing and phonatory dysfunctions, there has been a clear paradigm shift from radical surgery to organ-preserving CCRT for HPSCC treatment in recent decades.^3, 4^ The main concern of such an organ-preserving approach is whether definitive CCRT could provide comparable treatment outcomes compared with OP-CRT. Reviewing the literature, only two randomized control trials compared the treatment outcomes between surgery-based and radiation-based modalities before the popularity of CCRT.^8,9^ Unexpectedly, in the contemporary era of CCRT, no randomized trial has been conducted specifically on HPSCC patients to compare the treatment outcome between OP-CRT and CCRT.^6^ Retrospective studies that directly compared the outcome between CCRT and OP-CRT in HPSCC patients are also limited.^6,10^ The optimal management of HPSCC patients remains to be established.

Thus, this study provides our long-term experiences in treating HPSCC patients, comparing the survival outcomes and failure patterns between CCRT and OP-CRT treatment.

## Patients and Methods

### Demographic Data and Clinical Parameters

This study protocol was reviewed and approved by the Institutional Review Board (#B-ER-106-160) and informed consent could be waived to conduct this study. Patients with pathology proven primary HPSCC without distant metastasis evidence, who had completed a curative-intent treatment at National Cheng Kung University Hospital from 2007 to 2015, were included in this study. The demographic data and clinical parameters of these patients, including age, sex, lifestyle factors (smoking, drinking, and betel quid usage), TNM stage [7th edition American Joint Committee on Cancer (AJCC) staging system],^11^ treatment modalities, failure pattern, and survival outcomes were retrospectively obtained by retrieving data from the Institutional Cancer Registration Database, and/or from chart review. Patients treated between 2007 and 2009 were re-staged according to the 7th edition AJCC staging system. Patients with histological tumor type other than squamous cell carcinoma, with incomplete medical records, or with their hypopharyngeal cancer occurring as a second primary malignancy metachronous to index head and neck cancers, were excluded from the current study.

### Treatment Modalities

To decipher the effectiveness of surgery-based and radiation-based treatment for primary HPSCC, patients were further subdivided into two groups. For the CCRT group, the mainstay treatment was definitive concurrent chemoradiation or radiotherapy. For the OP-CRT group, curative surgery was the mainstay treatment, followed by adjuvant radiotherapy or chemoradiation. The treatment the patient receives is primarily based on the patient’s preference and the judgment of the treating physicians. Some cases received induction chemotherapy and/or adjuvant chemotherapy at the discretion of the primary treating physicians (including surgical oncologist, radiation oncologist and medical oncologist) and tumor board consensus according to the patient’s best benefit. In the surgical group, the most common cause for induction chemotherapy was the shortage of surgical delivery.^12^

### Radiotherapy Technique

An inverse planning software (Eclipse 13.6, Varian Medical Systems, Palo Alto, California) was used in all patients that received intensity-modulated radiation therapy (IMRT). A series of axial images (2.5- to 3.75-mm slices) were acquired from head to upper mediastinum by high resolution computed tomography (CT) simulator (Light-speed RT CT scanner, GE). The prescribed dose was 1.8–2 Gy/fraction/day given 5 days per week. Planning target volumes (PTVs) were created by the automated expansion of 3 to 5 mm of all clinical target volumes (CTVs) to account for setup errors. Normal structures, including the parotid glands, spinal cord, brain stem, optic nerves, and optic chiasm, were also contoured on the treatment plan. In the CCRT group, the high-risk clinical target volume (CTV-H) covered the gross hypopharyngeal tumor, adjacent larynx, tumor invasion areas, and involved nodes. The intermediate-risk CTV (CTV-M) included suspected nodes and neck levels with tumor involvement. The low-risk CTV (CTV-L) included all other low-risk lymphatic regions. Generally, neck levels Ib–VI and retropharyngeal nodal region were considered at-risk areas for occult micrometastases. The prescribed doses for the high-risk, intermediate-risk, and low-risk CTV/PTV were 66–70.2, 59.4–61.2, and 46–54 Gy, respectively. In the OP-CRT group, scheduled adjuvant radiotherapy was performed within 4–6 weeks after the operation if it was feasible. The CTV-H covered the primary tumor bed, soft tissue invasion areas, and areas of extra-nodal extension (ENE). The CTV-M included the entire surgical bed plus the involved neck levels without ENE. The CTV-L included all other lymphatic regions (levels Ib-IV) at lower risk for occult micrometastases. The prescribed radiation dose for CTV-H, CTV-M, and CTV-L was 66–66.6 Gy, 59.4–61.2 Gy, and 50–50.4 Gy, respectively. Patients with involved/close surgical margins might receive a higher dose at the discretion of the treating physician.

### Chemotherapy Regimen

For patients receiving either definitive or adjuvant chemoradiation, chemotherapy regimens mainly consisted of 3-weekly cisplatin (80–100 mg/m^2^) for 2–3 cycles,^13^ weekly cisplatin (40 mg/m^2^) for 5–6 cycles^14^ or cisplatin (20 mg/m^2^/day, days 1–4) plus fluorouracil (800 mg/m^2^/day, days 1–4) at 4-week intervals for 2 cycles during radiotherapy.^15^ In patients with renal insufficiency, hearing impairment, or age over 70, cetuximab was used as a radiosensitizer (Bio-RT).^16^ For patients receiving Bio-CRT, additional weekly cisplatin (30–40 mg/m^2^) for 5–6 cycles was administered.^17^ Induction chemotherapy was administered mainly with PF4, modified TPF, or PUL regimens. The PF4 regimen consisted of cisplatin (60–80 mg/m^2^ on day 1 or 20 mg/m^2^/day for days 1–4) plus fluorouracil (800 mg/m^2^/day, days 1–4) at 3-week intervals.^18^ Modified TPF regimens consisted of cisplatin (60 mg/m^2^ on day 1) plus docetaxel (60 mg/m^2^ on day 1) plus fluorouracil (1000 mg/m^2^/day on days 1–3) at 3-week intervals.^19^ The PUL regimen consisted of cisplatin (50 mg/m^2^ on day 1) plus oral tegafur-uracil (300 mg/m^2^/day on days 1–14) at 2-week intervals.^20^ Induction chemotherapy was administered for no more than 4 cycles before curative operation or (chemo-)radiation. Adjuvant chemotherapy was mainly conducted with oral tegafur-uracil 300 mg/m^2^/day for 1 year.^21^

### Post Treatment Assessment

After completing treatment, we regularly followed these HPC patients every 1–3 months in the first 2 years and every 4–6 months after that. Image study with either computed tomography or magnetic resonance imaging was performed every 3–6 months following treatment in the first 3 years. A biopsy was conducted if a patient was suspected of having a recurrent or persistent disease.

### Statistical Analysis

The difference between group variables was compared with either the chi-square test or Student’s *t*-test. Survivorship was analyzed using the Kaplan-Meier method. A log-rank test was used to compare the survivorships between treatment modalities. The hazard ratio and the associated 95% confidence interval were calculated using the Cox regression model. Statistical analyses were performed using SAS 9.2 (Cary, NC, USA) and R programming language (version 4.2.1) with R package ‘survival’ and ‘survminer.’ A probability value less than 0.05 (*p* < 0.05) was considered statistically significant.

The duration of time-to-event was calculated from the date of initiating curative treatment until documented events or censored at the date of the last follow-up if no events happened. Events for local relapse-free survival, regional relapse-free survival, loco-regional relapse-free survival, disease-free survival (DFS), and disease-specific survival (DSS) were local recurrence, neck recurrence, local and/or neck recurrence, local recurrence and/or regional recurrence and/or distant metastasis and disease-related death (caused by uncontrolled local, regional recurrence, or distant metastases), respectively. Overall survival (OS) denoted the survival period from the date of starting curative treatment to the date of death by any cause or last follow-up.

## Results

### Patient Characteristics and Treatment Modalities

A total of 198 primary HPSCC patients were included in this study. The demographic data and clinical parameters are shown in Table 1. More than 90% of patients had locally advanced diseases (stage III/IV) at diagnosis. Among these 198 patients, 136 patients were assigned to the CCRT group because surgery was not performed during primary treatment. The remaining 62 patients receiving primary curative surgery were assigned to the OP-CRT group. Table 1 shows that most of the clinical parameters were comparable between the two groups of patients. However, because patients with very advanced diseases (T4b, N3) were most likely to be considered poor candidates for curative operation by the surgeon, we noted that more T4b/N3 patients were assigned to the CCRT group (Table 1).

**Table 1:** Demographic data and clinical characteristics of patients (*n* = 198)

Characteristics	All patients (*n* = 198)		CCRT (*n* = 136)		OP-CRT (*n* = 62)		*p*^a^
	*N*	(%)	*N*	(%)	*N*	(%)	
*Age, mean (SE)*	54.73 (0.68)		54.99 (0.87)		54.15 (1.02)		0.56
≤ 70	183	(92.4)	123	(90.4)	60	(96.8)	0.20
> 70	15	(7.6)	13	(9.6)	2	(3.2)	
*Sex*							
Men	194	(98.0)	134	(98.5)	60	(96.8)	0.59
Women	4	(2.0)	2	(1.5)	2	(3.2)	
*Alcohol*							
No	42	(21.2)	26	(19.1)	16	(25.8)	0.27
Yes	154	(77.8)	109	(80.2)	45	(72.6)	
Unknown	2	(1.0)	1	(0.7)	1	(1.6)	
*Betel quid*							
No	66	(33.3)	44	(32.4)	22	(35.5)	0.63
Yes	130	(65.7)	91	66.9	39	62.9	
Unknown	2	(1.0)	1	(0.7)	1	(1.6)	
*Cigarette*							
No	72	(36.4)	51	(37.5)	21	(33.9)	0.65
Yes	124	(62.6)	84	61.8	40	64.5	
Unknown	2	(1.0)	1	(0.7)	1	(1.6)	
*Differentiation*							
Well	14	(7.1)	8	(5.9)	6	(9.7)	0.12
Moderate	114	(57.6)	77	(56.6)	37	(59.7)	
Poor	33	(16.7)	16	(11.8)	17	(27.4)	
Unknown	37	(18.7)	35	(25.7)	2	(3.2)	
*Subsite*							
Pyriform sinus	177	(89.4)	122	(89.7)	55	(88.7)	0.78
Postcricoid	7	(3.5)	4	(2.9)	3	(4.8)	
Posterior pharyngeal wall	14	(7.1)	10	(7.4)	4	(6.5)	
*Clinical T*							
1	17	(8.6)	11	(8.1)	6	(9.7)	0.07
2	38	(19.2)	30	(22.1)	8	(12.9)	
3	58	(29.3)	40	(29.4)	18	(29.0)	
4a	71	(35.9)	42	(30.9)	29	(46.8)	
4b	14	(7.1)	13	(9.6)	1	(1.6)	
*Clinical N*							
0	32	(16.2)	18	(13.2)	14	(22.6)	0.31
1+2a	38	(19.2)	26	(19.1)	12	(19.4)	
2b+2c	121	(61.1)	86	(63.2)	35	(56.4)	
3	7	(3.5)	6	(4.4)	1	(1.6)	
*Clinical stage*							
I	1	(0.5)	1	(0.7)	0	0	0.03
II	8	(4.0)	4	(2.9)	4	(6.5)	
III	28	(14.1)	18	(13.2)	10	(16.1)	
IVA	141	(71.2)	94	(69.1)	47	(75.8)	
IVB	20	(10.1)	19	(14.0)	1	(1.6)	

*SE* standard error

^a^*p*-values were calculated with the unknowns excluded

Because the best treatment modality for hypopharyngeal cancer has yet to be established, the choice of treatment was primarily based on the judgment of treating physicians and the preference of treatment for each patient. Detailed treatment modalities are provided in Table S1. In the CCRT group (*n* = 136), concurrent chemoradiation was the mainstay radiation-based treatment (120 patients, 88.2%), with only 5 (3.8%) patients receiving radiotherapy only. Bio-RT was given to 11 (8.1%) patients. Induction chemotherapy was performed in 45 (33.1%) patients before the initiation of curative radiation. After definitive CCRT, adjuvant chemotherapy was given to 15 (11.0%) patients. In the OP-CRT group (*n* = 62), 56 (90.3%) patients received total laryngopharyngectomy while the other six patients received open-neck partial laryngopharyngectomy.^22^ Before conducting curative surgery, a short course of induction chemotherapy was given to 17 (27.4%) patients. The mean and medium follow-up durations for patients without disease recurrence or death after treatment were 5.0 and 4.9 years, respectively.

### Outcome and Disease Status Following RT-Based Treatment

In the CCRT group (*n* = 136), at the 1-year follow-up, 65 patients (47.8%) remained free of disease, 68 (50%) had loco- and/or regional disease, and 3 (2.2%) patients had distant metastases without loco- and/or regional disease. Among the 68 patients with loco- and/or regional disease, 54 (79.4%) had persistent disease after treatment, with only 14 (20.6%) patients having a disease-free interval for more than 6 months. At the 2-year follow-up, 2 additional patients developed isolated distant metastases, and 8 developed loco- and/or regional recurrent disease. The 2-year disease-free rate was 40.4% (*n* = 55).

### Loco-Regional Relapse-Free Survival in OP-CRT Group vs CCRT Group

Organ preservation is the main concern of radiation-based treatment for hypopharyngeal cancer patients. We recommend performing salvage surgery for those who have failed definitive RT-based treatment, either in the form of persistent or recurrent disease in the loco-regional area, when patients’ general conditions permit, and they can accept the salvage operation as being against the original purpose of organ preservation. We first compared the primary 3-year local relapse-free survivorship between the CCRT and OP-CRT groups, stratified by T stage. In Table 2, our data clearly demonstrated a significant benefit for primary tumor control in the OP-CRT group compared with the CCRT group in patients with T3 or T4a disease (T3, 93 vs 48%, *p* < 0.0001; T4a, 88 vs 37%, *p* = 0.0005).

**Table 2:** Three-year local relapse-free survival stratified by T classification and regional relapse-free survival stratified by N classification

**T**	3 year local relapse-free survival			**N**	3 year regional relapse-free survival		
	CCRT (*n* = 136)	OP-CRT (*n* = 62)	*p*		CCRT (*n* = 136)	OP-CRT (*n* = 62)	*p*
1	0.90 (0.71–1.00)	1.00	0.29	0	0.76 (0.55–0.96)	0.85 (0.65–1.00)	0.54
2	0.70 (0.54–0.86)	1.00	0.001	1	0.74 (0.54–0.94)	0.89 (0.70–1.00)	0.29
3	0.48 (0.33–0.64)	0.93 (0.80–1.00)	< 0.0001	1+2a	0.77 (0.60–0.95)	0.89 (0.70–1.00)	0.36
4a	0.37 (0.22–0.53)	0.88 (0.75–1.00)	0.0005	2b+2c	0.60 (0.48–0.71)	0.93 (0.84–1.00)	< 0.0001
4b	0	N/A^a^	–^b^	3	N/A^a^	N/A^a^	–^b^

^a^Follow–up time < 3 years

^b^*p*-value was not calculated because the OP-CRT group had a follow-up time < 3 years

Nodal metastasis is recognized as the most important predictor for head and neck cancer patients.^23^ To investigate whether radiation-based therapy is comparable with the OP-CRT in controlling neck disease before employing salvage surgery, we subsequently compared the primary 3-year regional relapse-free survivals between the CCRT and OP-CRT groups stratified by the N stage. Table 2 clearly shows a superior neck control rate in the OP-CRT group compared with the CCRT group, especially in patients with advanced nodal disease (N2b + N2c, 93% vs 60%, *p* = 0.001).

Taken together, our data strongly supported that, compared with treatment with CCRT, the OP-CRT did result in significantly better local and regional control in primary hypopharyngeal cancer patients, especially in patients with advanced primary tumors (T3 and T4a) and/or advanced nodal (N2b + N2c) disease. Furthermore, for patients with stage IVA, OP-CRT provided better 3-year loco-regional relapse-free survival than CCRT (85% vs 37%, *p* < 0.0001).

### Distant Metastasis-Free Survival in OP-CRT Group vs CCRT Group

Distant metastasis remains one of the major causes of treatment failure in head and neck cancer patients, especially those with advanced-stage disease. Next, we compared the 3-year metastasis-free survival (i.e., distant control rate) in these two groups of patients stratified by clinical stage. As shown in Table 3, although only marginally significant, patients in the OP-CRT group tend to have more distant failures (hence lower distant control rate) in stage IVA diseases than patients in the CCRT group (62% vs 79%, *p* = 0.06).

**Table 3:** Three-year loco-regional relapse-free survival and distant metastasis-free survival stratified by clinical stage

Stage	3 year loco-regional relapse-free survival			Stage	3 year distant metastasis-free survival		
	CCRT (*n* = 136)	OP-CRT (*n* = 62)	*p*		CCRT (*n* = 136)	OP-CRT (*n* = 62)	*p*
I	1.00	N/A^a^	–^b^	I	1.00	N/A^a^	–^b^
II	1.00	0.71 (0.24–1.00)	0.24	II	1.00	1.00	**–**^b^
III	0.69 (0.46–0.92)	0.88 (0.67–1.00)	0.23	III	0.92 (0.76–1.00)	0.72 (0.39–1.00)	0.30
IVA	0.37 (0.27–0.47)	0.85 (0.75–0.96)	<0.0001	IVA	0.79 (0.69–0.88)	0.62 (0.47–0.77)	0.06
IVB	0.08 (0.00–0.23)	N/A^c^	–^d^	IVB	0.60 (0.35–0.86)	N/A^c^	–^d^

^a^*N* for stage I = 0 for the OP–CRT group

^b^*p*-value was not calculated because there was no event in both groups

^c^Follow–up time < 3 years

^d^*p*-value was not calculated because the OP–CRT group had a follow-up time < 3 years

### DFS, DSS and OS in OP-CRT Group vs CCRT Group

Table 4 shows the 3-year disease-free survival (DFS), 3-year disease-specific survival (DSS), and 3-year overall survival (OS) between these two groups of patients stratified by their clinical stage. It is noted that stage IVA patients in the OP-CRT group had significantly better 3-year DFS compared with patients in the CCRT group (56% vs 36%, *p* = 0.004), with marginally better 3-year DSS for the OP-CRT group (67% vs 50%, *p* = 0.07). The difference in 3-year OS in stage IVA patients between these two groups was insignificant (52% in the OP-CRT group vs 44% in the CRT group, *p* = 0.16). Table 5 shows the estimated 5-year DFS, 5-year DSS, and 5-year OS between the OP-CRT and CCRT groups.

**Table 4:** Three–year disease–free survival (DFS), disease–specific survival (DSS), and overall survival (OS) stratified by clinical stage

Stage	3 year disease–free survival			3 year disease–specific survival			3 year overall survival		
	CCRT (*n* = 136)	OP–CRT (*n* = 62)	*p*	CCRT (*n* = 136)	OP–CRT (*n* = 62)	*p*	CCRT (*n* = 136)	OP–CRT (*n* = 62)	*p*
I	1.00	N/A^a^	–^b^	1.00	N/A^a^	–^b^	1.00	N/A^a^	–^b^
II	1.00	0.71 (0.24–1.00)	0.24	1.00	1.00	–^b^	0.75 (0.33–1.00)	0.75 (0.33–1.00)	1.00
III	0.69 (0.46–0.92)	0.72 (0.39–1.00)	0.87	0.80 (0.59–1.00)	0.88 (0.67–1.00)	0.58	0.70 (0.48–0.92)	0.78 (0.51–1.00)	0.66
IVA	0.36 (0.26–0.46)	0.56 (0.41–0.71)	0.03	0.50 (0.40–0.61)	0.67 (0.52–0.81)	0.07	0.44 (0.34–0.54)	0.52 (0.38–0.67)	0.37
IVB	0.07 (0.00–0.19)	N/A^c^	–^d^	0.28 (0.07–0.49)	N/A^c^	–^d^	0.26 (0.07–0.46)	N/A^c^	–^d^

^a^*N* for stage I = 0 for the OP–CRT group

^b^*p*-value was not calculated because there was no event in both groups

^c^Follow–up time < 3 years

^d^*p*-value was not calculated because the OP–CRT group had a follow-up time < 3 years

**Table 5:** Five–year disease–free survival (DFS), disease–specific survival (DSS), and overall survival (OS) stratified by clinical stage

Stage	5year disease–free survival			5 year disease–specific survival			5 year overall survival		
	CCRT (*n* = 136)	OP–CRT (*n* = 62)	*p*	CCRT (*n* = 136)	OP–CRT (*n* = 62)	*p*	CCRT (*n* = 136)	OP–CRT (*n* = 62)	*p*
I	1.00	N/A^a^	–^b^	1.00	N/A^a^	–^b^	1.00	N/A^a^	–^b^
II	1.00	0.71 (0.24–1.00)	0.24	1.00	1.00	–^b^	0.45 (0.00–0.97)	0.75 (0.33–1.00)	0.31
III	0.69 (0.46–0.92)	0.72 (0.39–1.00)	0.87	0.80 (0.59–1.00)	0.88 (0.67–1.00)	0.58	0.40 (0.15–0.66)	0.39 (0.00–0.94)	0.96
IVA	0.32 (0.22–0.42)	0.53 (0.37–0.68)	0.03	0.43 (0.32–0.53)	0.60 (0.44–0.75)	0.09	0.31 (0.21–0.41)	0.44 (0.29–0.59)	0.15
IVB	0.07 (0.00–0.19)	N/A^c^	–^d^	0.15 (0.00–0.33)	N/A^c^	–^d^	0.14 (0.00–0.31)	N/A^c^	–^d^

^a^*N* for stage I = 0 for the OP–CRT group

^b^*p*-value was not calculated because there was no event in both groups

^c^Follow-up time < 5 years

^d^*p*-value was not calculated because the OP-CRT group had a follow-up time < 5 years

To provide the survivorships in the primary advanced, resectable hypopharyngeal patients, stage IVB (T4b, N3) patients were excluded for subsequent analysis because they were considered to have an extremely poor prognosis and were mostly assigned to receive radiation-based therapy instead of surgery (Table 1). After excluding stage IVB patients, the 3-year DSS and OS for patients with advanced, resectable diseases (stage III/IVA) in the OP-CRT group (*n* = 57) vs CCRT group (*n* = 112) were 70% vs 54% (*p* = 0.05) and 56% vs 48% (*p* = 0.32), respectively. The 5-year DSS and OS for patients with stage III/IVA in the OP-CRT group vs CCRT group were 64% vs 48% (*p* = 0.07) and 44% vs 32% (*p* = 0.18), respectively.

### Failure Pattern in OP-CRT Group vs CCRT Group

We also summarized the detailed cause of failure in these two groups of patients. Either ‘death (for any reason)’ or ‘alive with primary or second primary malignancy treated with palliative intent only’ were considered as ‘failure.’ After excluding stage IVB patients, we identified 77 patients in the CCRT group and 34 patients in the OP-CRT group as the target population considered ‘failure’ (Table S2). In the CCRT group, the major cause of failure was uncontrolled loco-regional disease (32 patients, 41.6%, *p* = 0.0006, compared with the OP-CRT group), followed by distant metastasis (13 patients, 16.9%). In contrast, the major cause of failure in the OP-CRT group was uncontrolled distant metastasis (13 patients, 38.2%, *p* = 0.01 compared with the CCRT group) rather than loco-regional relapse (3 patients, 2.9%). Failure caused by a second primary malignancy in the OP-CRT group was similar to that of the CCRT group (15.6% in the CCRT group vs 17.6% in the OP-CRT group).

### Survival Significance of Tracheostomy or Feeding Tube on HPC Patients

Among 136 patients undergoing CCRT, 26 patients received a tracheostomy, and 38 had a feeding tube (FT). Further subgroup analysis by tube types demonstrated that T4a/T4b patients were more likely to wear tracheostomy prior to or during radiotherapy (*n* = 17, 65.4%; *p* < 0.01; Table S3). In univariate analysis, DFS was significantly worse for patients with a tracheostomy than those without a tracheostomy (*p* = 0.041, Table S5). However, there was no statistically significant difference in overall survival (*p* = 0.6, Fig. S1A) and disease-specific survival between the groups (*p* = 0.52, Fig. S1B). In contrast, patients with FTs had poorer DFS (*p* <0.001, Table S5) and OS (*p* = 0.0026, Fig. S2A) than those without FTs. Similarly, DSS was significantly worse for patients with FTs than those without (*p* = 0.015, Fig. S2B), despite a trend toward advanced T classification (*p* = 0.06, Table S4) and late stage (*p* = 0.07, Table S4) in patients managed with a feeding tube. Exploratory multivariate analysis by tube categories for DFS showed feeding tube placement was marginally predictive for poor DFS (*p* = 0.058, Table S5); however, tracheostomy wearing was not identified as the predictive factor for DFS (*p* = 0.399, Table S5).

## Discussion

The optimal management for advanced hypopharyngeal cancer patients remains undetermined. In the pre-CCRT era, according to the results from the two important randomized control trials conducted on advanced hypopharyngeal cancer patients,^8,9^ the surgery-based treatment seems to have better treatment results in both disease control and patient survival when compared with radiation-based treatment. In one of these two trials, Beauvillain et al.^9^ compared the treatment results in 90 resectable hypopharyngeal cancer patients (T3–4, N0–3) by dividing them into two groups: induction chemotherapy plus radiotherapy group (*RT* arm) vs induction chemotherapy plus surgery plus radiotherapy group (*S+RT* arm). In this trial, more than 80% of patients in both groups first completed 3 cycles of induction chemotherapy as scheduled. Although fewer patients in the *S+RT* arm (67%) achieved complete or partial response after induction chemotherapy than patients in the *RT* arm (79%), the 5-year local control (63% vs 39%, *p* < 0.01) and 5-year OS (37% vs 19%, *p* = 0.04) were significantly better in the *S+RT* arm. Notably, in both groups, chemotherapy responders also had better OS than non-responders. Another phase III clinical trial^8^ from the EORTC Head and Neck Cancer Cooperative Group was conducted to test the feasibility of laryngeal preservation by a non-surgical approach in 202 stage II–IV (T2–4, N0–3) resectable pyriform sinus cancer patients. Patients were first randomized into the immediate surgery group or induction chemotherapy group in this trial. Patients in the immediate surgery group received total laryngectomy + partial pharyngectomy + radiotherapy, while patients in the induction chemotherapy group received 3 scheduled cycles of chemotherapy (cisplatin 100 mg/m^2^ + 5-FU 1000 mg/m^2^) first (*CT* arm). In the *CT* arm, only patients who achieved complete response after two or three cycles of chemotherapy proceeded to definitive radiotherapy. After induction chemotherapy selection, 35% of patients in the *CT* arm proceeded to curative surgery instead of definitive radiotherapy. After radiotherapy, a salvage operation had to be performed in 12 additional patients (8 to primary and 4 to the neck) in the *CT* arm. Although both the 5-year disease-free survival and overall survival were comparable in both arms, considering that: (1) 35% of patients in the *CT* arm did not achieve complete response after chemotherapy (about half of T3 patients and all of the T4 patients in the *CT* arm had to receive curative surgery rather than proceeding to subsequent radiotherapy), and (2) salvage operation was performed in 8 additional patients with local failure following radiotherapy in the *CT* arm, it is reasonable to conclude that primary surgery (followed by radiotherapy) would provide superior local disease control and better survival compared with primary radiotherapy alone if pre-radiotherapy chemotherapy selection were not performed.

In the contemporary era, however, cumulating evidence has demonstrated that IMRT may confer better loco-regional control in head and neck cancers compared with conventional radiotherapy.^24^ The administration of chemotherapy with IMRT may further improve disease control for patients with locally advanced head and neck squamous cell carcinoma.^25^ Thus, although there’s still no randomized trial comparing the effectiveness between surgery-based treatment and the radiation-based approach (with chemotherapy) in locally advanced hypopharyngeal cancers, it is natural to postulate that, with the advancement of radiotherapy technology and the addition of chemotherapy, the local disease control advantage and the survival benefit from primary surgery noted in the pre-CCRT era would become insignificant compared with contemporary organ-preservation CCRT. In addition, radiation-based therapy could provide the opportunity for organ preservation in advanced T staged patients who will inevitably require extensive resection of laryngeal and hypopharyngeal structures. Encouraged by the results from an organ-preserving trial conducted on advanced laryngeal cancers,^26^ in recent decades, there has also been a clear trend of changing from surgery-based approach toward nonsurgical radiation-based therapy for hypopharyngeal cancer patients, even in patients with advanced T stage diseases.^2–4^

Results from our study are somewhat against the assumption that definitive CCRT will provide equal loco-regional disease control compared with OP-CRT. Our results clearly demonstrated that, compared with the CCRT approach, there are still significant local and regional control advantages in advanced T (T3, T4a) or advanced N stage (N2b, N2c) patients receiving OP-CRT as their primary treatment (Table 2). In support of our findings, Lee et al.^27^ also reported a better local disease control rate in the surgery plus postoperative radiotherapy group than in the CCRT (*p* < 0.05). With a match-paired analysis conducted on advanced hypopharyngeal cancer patients from multiple institutions (*n* = 254), Iwae et al.^28^ also demonstrated a significantly better 5-year loco-regional control rate in the total pharyngolaryngectomy group compared with the chemoradiation group (82.2% vs 63.6%, *p* < 0.01). Thus, even in the era of CCRT, it seems that surgery-based primary treatment still offers better local and regional disease control in advanced-stage hypopharyngeal cancer patients than those treated with definitive CCRT.

Unexpectedly, in our study, although advance-staged patients in the OP-CRT group had fewer loco-regional recurrences (Table 3), we did notice that a higher proportion of stage IVA patients in the OP-CRT group suffered from distant metastasis after treatment compared with patients in the CCRT group (*p* = 0.06). The underlying cause for this observation may be explained by two speculations: (1) fewer patients in the OP-CRT group received a full course of induction chemotherapy before curative surgery, and (2) more patients in the CCRT group died from uncontrolled loco-regional disease before distant metastasis became clinically evident. Notably, the EORTC trial^8^ also cautiously reported a higher distant metastasis rate observed in the primary surgery arm (36%) than in the *CT* arm (25%).

One strength of our study is that, by providing a detailed failure pattern analysis on local, regional, and distant sites after treatment, we were able to provide additional insights to interpret the survival data in our patients. For instance, in advanced-stage patients (especially in stage IVA), although we noticed a clear, inarguable advantage of the 3-year loco-regional relapse-free survival in the OP-CRT group (stage IVA, *p* < 0.0001, Table 3), the difference in 3-year disease-free survival between these two groups was less remarkable (stage IVA, *p* = 0.03, Table 4) due to more distant metastasis in the OP-CRT group (Table 3). And since curative intent salvage treatment for distant failure (more patients in the OP-CRT group) is less likely to succeed than salvage treatment for loco-regional relapse (more patients in the CCRT group), we only noticed a marginally significant benefit of 3-year DSS in the OP-CRT group compared with the CCRT group (stage IVA, 67 vs 50%, *p* = 0.07, Table 4). Considering that OS may additionally be attributed to factors unrelated to primary treatment, the actual benefit in a specific treatment modality might be obscured in studies that cannot recruit a large number of patients. Our study noted that 30–40% of ‘failure’ was caused by either a second primary tumor or disease-unrelated death in both groups of patients (Table S2). Thus, it is not unexpected to see that the difference in the 3-year OS would become statistically non-significant (52 vs 44%, *p* = 0.37, Table 4) between these two groups. Taken together, our study advocates that, when comparing different treatment modalities, each survival data should be interpreted cautiously.

Based on the above understandings, it is not surprising that some of the previous studies conducted on advanced-stage hypopharyngeal cancer patients reported better local or loco-regional control rates in the primary surgery group but failed to demonstrate significantly better DSS or OS compared with the CCRT.^27,28^ It is also reasonable to see that, in retrospective cohort studies comparing DSS or OS between primary surgery and definitive chemoradiation, most studies reported marginally better results, or a trend toward better results, in the surgery group than in the radiation group. For instance, Tsou et al.^29^ reported better 5-year DSS in the primary surgery followed by CCRT group compared with the primary CCRT followed by early surgical salvage group in both stage III (51 vs 38%, *p* = 0.03) and stage IV (23.1 vs 11%, *p* = 0.05) patients. Kim et al.^30^ reported non-statistically significant improvements in the 5-year DFS (64 vs 51%, *p* = 0.49) and OS (67% vs 59%, *p* = 0.83) in resectable stage III/IV patients receiving total laryngectomy compared with patients receiving definitive CCRT. Harries et al.^10^ reported a trend toward improvement in 5-year relapse-free survival (RFS) (41% vs 35%, *p* = 0.18) and OS (66 vs 54%, *p* = 0.09) in advanced-stage patients receiving primary surgery compared with definitive CCRT. After multivariate Cox regression analysis, that study similarly showed a trend toward improved RFS (hazard ratio 2.97, *p* = 0.12) and OS (hazard ratio 4.78, *p* = 0.06) in patients receiving surgery.^10^ In a population-based study, Cheng et al.^31^ reported OS benefits in both stage III and stage IVA patients receiving primary surgery. Another population-based study similarly reported a better 5-year OS in T4 patients receiving total laryngectomy than patients receiving definitive CCRT (29% vs 25%, *p* = 0.04).^4^ In contrast, Jang et al.^32^ demonstrated comparable 5-year OS for both primary surgery and definitive CCRT groups in 177 resectable, advanced-stage hypopharyngeal cancer patients. In a population-based study, Kim et al.^33^ failed to demonstrate a 3-year OS advantage in the surgery group compared with the definitive CCRT group, even in T4a patients (30 vs 26%, *p* = 0.44). The above evidence implies that, while the addition of surgery could confer better loco-regional control in advanced-stage hypopharyngeal cancer patients, the survival advantage of better loco-regional control in the surgery group may not be directly translated into better DFS, DSS, or OS, due to diverse clinical conditions specific to the included study population. Thus, randomized control trials are mandatory to decipher the best approach for locally advanced hypopharyngeal cancer patients.

Another strength of our study is that, by providing a detailed analysis of the failure patterns, we were able to improve our treatment strategies accordingly. For patients with early stage disease (stages I, II), effective disease control can be achieved using either surgery or radiotherapy. Thus, a single modality treatment, such as laryngeal conservation surgery or (chemo-)radiotherapy,^34^ will be preferred to minimize treatment-related morbidity and maximize functional preservation. For patients with advanced-stage disease who choose to have a primary operation, induction chemotherapy or adjuvant chemotherapy should be considered to decrease the probability of distant metastasis that might occur after curative treatment, especially in T4a, N2b, and N2c patients. For patients who choose definitive CCRT as their primary treatment, we may perform chemotherapy selection^8,35^ to identify good responders before curative radiation to minimize the chance of difficult salvage surgery after CCRT.^34^ For patients with very advanced-stage disease (T4b or N3), clinical trials with novel therapeutic approaches may be considered a priority.

Previous investigations have demonstrated the survival significance of tracheostomy and feeding tubes on the survival of patients undergoing (chemo)radiotherapy. In terms of tracheostomy, an earlier retrospective study of 270 irradiated laryngeal cancer patients demonstrated that patients without tracheostomy have better 2-year disease-free survival than those with tracheostomy (74% vs 41%).^36^ The loco-regional controls differ by anatomic subsite, with the most significant difference occurring in the glottis (tracheostomy vs no-tracheostomy: 69% vs 94%). Of interest, almost all the patients wearing tracheostomy had T3/T4-stage lesions, reflecting the tracheostomy as a surrogate for locally advanced tumors with airway comprises. Later, in a single-institution study investigating the factor predicting organ preservation failure after CCRT, Heukelom et al.^37^ also identified T4 lesion and pretreatment tracheostomy as independent prognostic factors in laryngeal and hypopharyngeal cancers. Our results are consistent with these findings that the tracheostomy prior to, or during, the radiotherapy may be a surrogate for an advanced T stage. In addition, patients without tracheostomy did show statistically significant oncologic benefits over patients without tracheostomy, although tracheostomy was not identified as an independent prognostic factor for DFS after controlling for other variables. On the other hand, enormous investigations have emphasized the significance of feeding tubes [mainly percutaneous endoscopic gastrostomy (PEG)] in laryngo- and pharyngeal-cancer patients treated with CCRT. A systematic review conducted by Mellors et al.^38^ demonstrated that the prophylactic PEG was associated with reduced incidence of critical weight loss (> 10% body weight loss) and improved short–term quality of life compared with those receiving reactive nutritional support. The results are consistent with the review by McClelland et al.^39^, supporting the concept of prophylactic PEG placement in patients at risk for malnutrition during radiation therapy. However, our analysis demonstrated that feeding tube use was negatively associated with disease-free survival, disease-specific survival, and overall survival. We hypothesize that the negative impact was that some of our patients in the CCRT group received reactive feeding tubes instead. The inferior survival in the feeding tube group may be because the patients with reactive nutritional support have already developed significant weight loss^40^ or poorer tolerance^41^ to radiation that causes RT interruption or prolongation, which are recognized as major negative prognostic factors for patients receiving (chemo)radiotherapy. Consequently, despite the limited recruited patients and the retrospective design in the current study, our conclusions imply that execution of the upfront surgery rather than CCRT on patients needing FT may improve the overall prognosis of hypopharyngeal cancer. However, a well-designed prospective study is needed to confirm our hypothesis in the future.

There are some limitations to our study. First, the treatment modality used for the OP-CRT and the CCRT groups was not uniform (Table S1). Second, whether the genetic background of each study population impacts the prognosis related to each specific treatment modality (primary surgery vs radiation) is not known. It is noticeable that the other two studies also from Taiwan similarly reported favorable outcomes in advanced-stage hypopharyngeal cancer patients receiving primary surgery.^29,31^ We also recently demonstrated that pre-diagnosis alcohol consumption and the polymorphisms of ALDH2 impact the prognosis of HNC.^42^ In a recent study conducted in Japan, heavy drinking combined with the ALDH2*2 allele was an independent poor prognostic factor of OS and DFS for hypopharyngeal cancer patients.^43^ Since the population in East Asia, including Taiwan and Japan, has the highest prevalence of the poor functional allele (ALDH2*2) in the world,^44^ the prognostic impact of ALDH2 polymorphism on hypopharyngeal cancer treatment deserves further investigation. Third, the functional results of speech, swallowing, and quality life assessment in our study population require additional study. Knowledge of the nutrition impact symptoms, functional disabilities, and potential morbidity profiles by different treatments are important because some studies have established their associations with quality of life^45^ and survival^46^ in head and neck cancer patients. However, the relevant information in the current study is insufficient. Further study is warranted for metrics measurements by different therapeutic strategies to refine our treatment delivery for each HPC patient. Fourth, we have few elders in the current study, which limited the analysis regarding impact of age on survival. Elderly patients are more vulnerable to developing adverse events after interventions.^47^ Therefore, the best choice of treatment and whether to treat or not should consider many aspects because the associated complications may potentially translate into a higher proportion of complication-related deaths. Fifth, no randomized trial has been conducted to compare the outcome difference between patients receiving CCRT or induction chemotherapy (IC) followed by CRT. However, a recent retrospective study by Chung et al.^48^ found their 127 staged III/IV HPC patients have comparable 5-year overall survival, disease-free survival after salvages, and laryngeal preservation rates between the two treatments, supporting our methodology by merging CCRT and IC+CRT for further analysis. However, future solid evidence is needed to identify the factors that benefit from either treatment, especially for the patients proceeding with an organ preservation strategy. Sixth, all patients in the current investigation were staged according to the definitions outlined in the AJCC 7th edition because we performed data collection before the general adoption of the AJCC 8th edition in 2018. The major difference between the 8th edition and the 7th is the separation of hypopharyngeal cancer from pharynx cancer and the categorization of the involved lymph node to N3b if an extra-nodal extension (ENE) was present.^49^ However, Chai et al.^50^ reported that using contrast-enhanced computed tomography alone to diagnose ENE of regional lymph nodes is not very reliable. Because there is no pathologic evidence for the CCRT group, re-classification from the 7th to 8th edition may lead to mal-categorization, resulting in the misinterpretation of our results. Another limitation is that the time-to-event is a methodologically relevant outcome in a retrospective cohort. However, we hypothesize that the effect should be minimized in the current study because the evaluation modalities are generally uniform before and after the primary treatments. Additionally, we have recommended a more vigilant post-treatment surveillance for patients with a high risk for recurrence and relapse. As for the clinically controversial cases, we will actively arrange a biopsy of the suspicious lesion to confirm the pathologic diagnosis. If the patient declined a biopsy, we reached a consensus through discussions with experts in other fields. Lastly, knowledge regarding hospital admission stay is a specific issue in oncology because of its significant impacts on the health care delivery policy and patients’ health-related quality of life.^51^ With data from the National Health Insurance program in Taiwan, we might decipher the most optimal treatment strategy for this devastating disease by integrating the analysis of healthcare costs in different treatment modalities.

## Conclusions

In conclusion, our study clearly demonstrated that, for advanced-stage hypopharyngeal cancer patients (especially T3–4a and N2b–c), primary surgery followed by adjuvant (chemo-)radiotherapy (OP-CRT) provides better local and regional disease control compared with definitive chemoradiation (CCRT). However, a higher distant metastasis rate was noted in the OP-CRT group. The detailed analysis of failure patterns will pave the way for improving treatment strategies for this devastating disease.

## Supplementary Information

Below is the link to the electronic supplementary material.

Supplementary file1 (DOCX 732 kb)

## Data Availability

The datasets used and/or analyzed during the current study are available from the corresponding author on reasonable request.
