# Supplementary material for: Distinct Failure Patterns in Hypopharyngeal Cancer Patients Receiving Surgery-Based Versus Radiation-Based Treatment
Source: Ann Surg Oncol. 2022 Nov 6;30(2):1169–81. doi: 10.1245/s10434-022-12744-1 (PMC9807465; doi:10.1245/s10434-022-12744-1)

**Supplementary documentation**

**Supplementary Tables**

**Table S1. Treatment modalities in CCRT and OP-CRT groups**

| CCRT (*n* = 136) | | | OP-CRT (*n* = 62) | | | |
| --- | --- | --- | --- | --- | --- | --- |
| Treatment Modality | *N* | % |  | Treatment Modality | *N* | % |
| RT | 5 | 3.7 |  | Op | 5 | 8.1 |
| CRT | 69 | 50.7 |  | Op+ RT | 4 | 2.9 |
| CRT+ CT | 11 | 8.1 |  | Op+ CRT | 35 | 56.5 |
| Induction CT+ CRT | 37 | 27.2 |  | Op+ CRT+ CT | 1 | 1.6 |
| Induction CT+ CRT+ CT | 3 | 2.2 |  | Induction CT+ Op | 7 | 11.3 |
| Bio-RT | 6 | 4.4 |  | Induction CT+ Op+ CT | 1 | 1.6 |
| Induction CT+ RT | 0 | - |  | Induction CT+ Op+ RT | 2 | 3.2 |
| Induction CT+ Bio-RT | 2 | 1.5 |  | Induction CT+ Op+ CRT | 7 | 11.3 |
| Induction CT+ Bio-RT+ CT | 1 | 0.7 |  |  |  |  |
| Induction CT+ Bio-CRT | 1 | 0.7 |  |  |  |  |
| Induction CT+ Bio-CRT+ CT | 1 | 0.7 |  |  |  |  |

*RT* radiotherapy, *CRT* concurrent chemoradiation, *CT* chemotherapy, *Op* operation,

*Bio-RT* cetuximab + radiotherapy,

*Bio-CRT* cetuximab + chemoradiation

**Table S2. Cause of ‘failure’ in the CCRT vs. OP-CRT group**

|  | CCRT*  (*n* = 77) | |  | OP-CRT*  (*n* = 34) | |  |
| --- | --- | --- | --- | --- | --- | --- |
| Cause of failure | *N* | % |  | *N* | % | *p* |
| L or R or LR | 32 | 41.6 |  | 3 | 2.9 | 0.0006 |
| DM | 13 | 16.9 |  | 13 | 38.2 | 0.01 |
| DM + L or R or LR | 5 | 6.5 |  | 5 | 14.7 | 0.16 |
| Second primary malignancy | 12 | 15.6 |  | 6 | 17.6 | 0.79 |
| Death unrelated to above cause | 15 | 19.5 |  | 7 | 20.6 | 0.89 |

* Stage IVB excluded

*L* local recurrence, *R* regional recurrence, *LR* loco-regional recurrence,

*DM* distant metastasis

**Table S3. Clinical Characteristics of CCRT group by tracheostomy status**

|  | No tracheostomy  (*n* = 110) | | With Tracheostomy  (*n* = 26) | |  |
| --- | --- | --- | --- | --- | --- |
| Characteristics | *N* | % | *N* | % | *p*^a^ |
| Age, mean (SE) | 54.6 (0.98) | | 56.7 (1.93) | | 0.35 |
| Sex |  |  |  |  |  |
| Men | 108 | 98.2 | 26 | 100 | 1.00 |
| Women | 2 | 1.8 | 0 | 0 |  |
| Alcohol |  |  |  |  |  |
| No | 19 | 17.3 | 7 | 26.9 | 0.41 |
| Yes | 90 | 81.8 | 19 | 73.1 |  |
| Unknown | 1 | 0.9 | 0 | 0 |  |
| Betel quid |  |  |  |  |  |
| No | 35 | 31.8 | 9 | 34.6 | 0.99 |
| Yes | 74 | 67.3 | 17 | 65.4 |  |
| Unknown | 1 | 0.9 | 0 | 0 |  |
| Cigarette |  |  |  |  |  |
| No | 39 | 35.5 | 12 | 46.2 | 0.45 |
| Yes | 70 | 63.6 | 14 | 53.8 |  |
| Unknown | 1 | 0.9 | 0 | 0 |  |
| Differentiation |  |  |  |  |  |
| Well | 6 | 5.5 | 2 | 7.7 | 0.10 |
| Moderate | 60 | 54.5 | 17 | 65.4 |  |
| Poor | 11 | 10 | 5 | 19.2 |  |
| Unknown | 33 | 30 | 2 | 7.7 |  |
| Subsite |  |  |  |  |  |
| Pyriform sinus | 97 | 88.2 | 25 | 96.2 | 0.44 |
| Postcricoid | 4 | 3.6 | 0 | 0 |  |
| Posterior pharyngeal wall | 9 | 8.2 | 1 | 3.8 |  |
| Clinical T |  |  |  |  |  |
| 1/2 | 39 | 35.5 | 2 | 7.7 | < 0.01 |
| 3 | 33 | 30 | 7 | 26.9 |  |
| 4a/4b | 38 | 34.5 | 17 | 65.4 |  |
| Clinical N |  |  |  |  |  |
| 0 | 14 | 12.7 | 4 | 15.4 | 0.58 |
| 1/2a | 22 | 20 | 4 | 15.4 |  |
| 2b/2c | 68 | 61.8 | 18 | 69.2 |  |
| 3 | 6 | 5.5 | 0 | 0 |  |
| Clinical stage |  |  |  |  |  |
| I-III | 21 | 19.1 | 2 | 7.7 | 0.17 |
| IVA | 76 | 69.1 | 18 | 69.2 |  |
| IVB | 13 | 11.8 | 6 | 23.1 |  |

*CCRT* concurrent chemoradiotherapy, *SE* standard error

^a^ *p*-values were calculated with the unknowns excluded

**Table S4. Clinical Characteristics of CCRT group by feeding tube status**

|  | No Feeding Tube  (*n* = 98) | | | Feeding Tube  (*n* = 38) | | |  |
| --- | --- | --- | --- | --- | --- | --- | --- |
| Characteristics | *N* | % | *N* | | % | *p*^a^ | |
| Age, mean (SE) | 54.3 (0.98) | | 56.8 (1.81) | | | 0.19 | |
| Sex |  |  |  | |  |  | |
| Men | 97 | 99.0 | 37 | | 97.4 | 1.00 | |
| Women | 1 | 1.0 | 1 | | 2.6 |  | |
| Alcohol |  |  |  | |  |  | |
| No | 18 | 18.4 | 8 | | 21.1 | 0.85 | |
| Yes | 80 | 81.6 | 29 | | 76.3 |  | |
| Unknown | 0 | 0 | 1 | | 2.6 |  | |
| Betel quid |  |  |  | |  |  | |
| No | 27 | 27.6 | 17 | | 44.7 | 0.07 | |
| Yes | 71 | 72.4 | 20 | | 52.6 |  | |
| Unknown | 0 | 0 | 1 | | 2.6 |  | |
| Cigarette |  |  |  | |  |  | |
| No | 41 | 41.8 | 10 | | 26.3 | 0.17 | |
| Yes | 57 | 58.2 | 27 | | 71.1 |  | |
| Unknown | 0 | 0 | 1 | | 2.6 |  | |
| Differentiation |  |  |  | |  |  | |
| Well | 7 | 7.1 | 1 | | 2.6 | 0.60 | |
| Moderate | 53 | 54.1 | 24 | | 63.2 |  | |
| Poor | 11 | 11.2 | 5 | | 13.2 |  | |
| Unknown | 27 | 27.6 | 8 | | 21.1 |  | |
| Subsite |  |  |  | |  |  | |
| Pyriform sinus | 90 | 91.8 | 32 | | 84.2 | 0.39 | |
| Postcricoid | 2 | 2.0 | 2 | | 5.3 |  | |
| Posterior pharyngeal wall | 6 | 6.1 | 4 | | 10.5 |  | |
| Clinical T |  |  |  | |  |  | |
| 1/2 | 35 | 35.7 | 6 | | 15.8 | 0.06 | |
| 3 | 28 | 28.6 | 12 | | 31.6 |  | |
| 4a/4b | 35 | 35.7 | 20 | | 52.6 |  | |
| Clinical N |  |  |  | |  |  | |
| 0 | 14 | 14.3 | 4 | | 10.5 | 0.46 | |
| 1/2a | 21 | 21.4 | 5 | | 13.2 |  | |
| 2b/2c | 58 | 59.2 | 28 | | 73.7 |  | |
| 3 | 5 | 5.1 | 1 | | 2.6 |  | |
| Clinical stage |  |  |  | |  |  | |
| I-III | 21 | 21.4 | 2 | | 5.3 | 0.07 | |
| IVA | 65 | 66.3 | 29 | | 76.3 |  | |
| IVB | 12 | 12.2 | 7 | | 18.4 |  | |

*CCRT* concurrent chemoradiotherapy, *SE* standard error

^a^ *p*-values were calculated with the unknowns excluded

**Table S5. Univariate and Multivariate Cox Regression Analysis for Disease Free Survival in the CCRT group**

|  |  |  | Univariate | | |  | Multivariate (tracheostomy) | | | | |  | |  | | Multivariate (feeding tube) | | | | |
| --- | --- | --- | --- | --- | --- | --- | --- | --- | --- | --- | --- | --- | --- | --- | --- | --- | --- | --- | --- | --- |
| **Characteristics** |  | Event | HR | 95% CI | *p*-value |  | | HR | 95% CI | *p*-value |  | |  | | HR | | 95% CI | | *p*-value |  |
| Alcohol^a^ |  |  |  |  |  |  | |  |  |  |  | |  | |  | |  |  | |  |
| No (*n* = 26) |  | 17 | 1 |  |  |  | |  |  |  |  | |  | |  | |  |  | |  |
| Yes (*n* = 109) |  | 70 | 1.07 | 0.63-1.82 | 0.807 |  | |  |  |  |  | |  | |  | |  |  | |  |
| Betel quid^a^ |  |  |  |  |  |  | |  |  |  |  | |  | |  | |  | |  |  |
| No (*n* = 44) |  | 31 | 1 |  |  |  | |  |  |  |  | |  | |  | |  | |  |  |
| Yes (*n* = 91) |  | 56 | 0.79 | 0.51-1.23 | 0.300 |  | |  |  |  |  | |  | |  | |  | |  |  |
| Cigarette^a^ |  |  |  |  |  |  | |  |  |  |  | |  | |  | |  | |  |  |
| No (*n* = 51) |  | 32 | 1 |  |  |  | |  |  |  |  | |  | |  | |  | |  |  |
| Yes (*n* = 84) |  | 55 | 1.15 | 0.74-1.78 | 0.539 |  | |  |  |  |  | |  | |  | |  | |  |  |
| Differentiation |  |  |  |  |  |  | |  |  |  |  | |  | |  | |  | |  |  |
| Well (*n* = 8) |  | 5 | 1.00 |  |  |  | |  |  |  |  | |  | |  | |  | |  |  |
| Moderate (*n* = 77) |  | 55 | 1.13 | 0.45-2.82 | 0.801 |  | |  |  |  |  | |  | |  | |  | |  |  |
| Poor (*n* = 16) |  | 10 | 1.04 | 0.35-3.04 | 0.945 |  | |  |  |  |  | |  | |  | |  | |  |  |
| Unknown (*n* = 35) |  | 18 | 0.63 | 0.23-1.70 | 0.363 |  | |  |  |  |  | |  | |  | |  | |  |  |
| Clinical T |  |  |  |  |  |  | |  |  |  |  | |  | |  | |  | |  |  |
| T1-2 (*n* = 41) |  | 21 | 1 |  |  |  | | 1 |  |  |  | |  | | 1 | |  | |  |  |
| T3 (*n* = 40) |  | 24 | 1.51 | 0.84-2.71 | 0.170 |  | | 1.63 | 0.89-2.99 | 0.115 |  | |  | | 1.51 | | 0.82-2.78 | | 0.187 |  |
| T4a-4b (*n* = 55) |  | 43 | 2.19 | 1.30-3.70 | < 0.01 |  | | 1.75 | 0.90-3.42 | 0.099 |  | |  | | 1.67 | | 0.87-3.18 | | 0.122 |  |
| Clinical N |  |  |  |  |  |  | |  |  |  |  | |  | |  | |  | |  |  |
| N0 (*n* = 18) |  | 7 | 1 |  |  |  | | 1 |  |  |  | |  | | 1 | |  | |  |  |
| N1/2a (*n* = 26) |  | 13 | 1.46 | 0.58-3.66 | 0.422 |  | | 1.19 | 0.45-3.19 | 0.725 |  | |  | | 1.24 | | 0.47-3.32 | | 0.665 |  |
| N2b/2c (*n* = 86) |  | 62 | 2.33 | 1.06-5.09 | 0.034 |  | | 1.39 | 0.52-3.69 | 0.511 |  | |  | | 1.41 | | 0.55-3.63 | | 0.475 |  |
| N3 (*n* = 6) |  | 6 | 5.72 | 1.90-17.28 | < 0.01 |  | | 3.62 | 0.91-14.36 | 0.067 |  | |  | | 3.58 | | 0.92-13.86 | | 0.065 |  |
| Clinical stage |  |  |  |  |  |  | |  |  |  |  | |  | |  | |  | |  |  |
| I-III (*n* = 23) |  | 6 | 1 |  |  |  | | 1 |  |  |  | |  | | 1 | |  | |  |  |
| IVA (*n* = 94) |  | 65 | 3.41 | 1.47-7.88 | < 0.01 |  | | 2.30 | 0.75-7.06 | 0.145 |  | |  | | 2.03 | | 0.68-6.02 | | 0.202 |  |
| IVB (*n* = 19) |  | 17 | 7.52 | 2.93-19.28 | < 0.001 |  | | 3.63 | 0.99-13.3 | 0.052 |  | |  | | 3.29 | | 0.92-11.83 | | 0.068 |  |
| Tracheostomy^b^ |  |  |  |  |  |  | |  |  |  |  | |  | |  | |  | |  |  |
| No (*n* = 110) |  | 67 | 1 |  |  |  | | 1 |  |  |  | |  | |  | |  | |  |  |
| Yes (*n* = 26) |  | 21 | 1.67 | 1.02-2.72 | 0.041 |  | | 1.28 | 0.72-2.26 | 0.399 |  | |  | |  | |  | |  |  |
| Feeding tube^b^ |  |  |  |  |  |  | |  |  |  |  | |  | |  | |  | |  |  |
| No (*n* = 98) |  | 56 | 1 |  |  |  | |  |  |  |  | |  | | 1 | |  | |  |  |
| Yes (*n* = 38) |  | 32 | 2.15 | 1.39-3.33 | < 0.001 |  | |  |  |  |  | |  | | 1.57 | | 0.99-2.51 | | 0.058 |  |

*CCRT* concurrent chemoradiotherapy*, HR* hazard ratio, *CI* confidence interval

^a^ *p*-values was calculated with the unknown excluded

^b^ Having tracheostomy or feeding tube before or during CCRT

**Supplementary Figures**

**Figure S1. Kaplan–Meier curves demonstrate the OS and DSS for CCRT patients with or without tracheostomy.**

(A) Patients with tracheostomy had comparable OS to those without (*p* = 0.6). (B) No difference in DSS among the two different tracheostomy groups (*p* = 0.52).

*CCRT* concurrent chemoradiotherapy, *OS* overall survival, *DSS* disease-specific survival


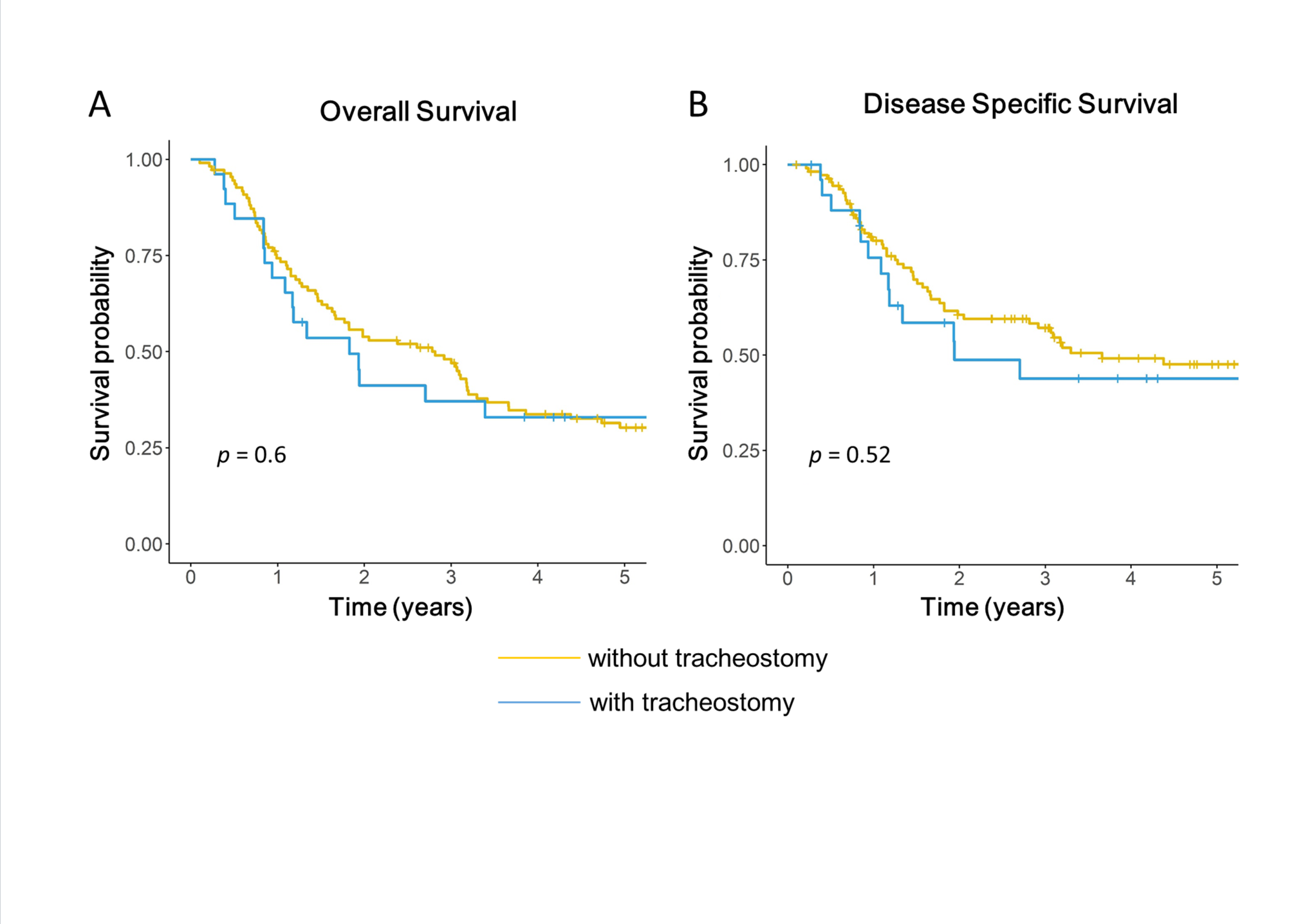


**Figure S2. The OS and DSS for CCRT patients by feeding tube status.**

(A) Patients with feeding tubes had worse OS (*p* = 0.0026). (B) The difference in DSS among patients with- and without feeding tubes was statistically significant (*p* = 0.015).

*CCRT* concurrent chemoradiotherapy, *OS* overall survival, *DSS* disease-specific survival


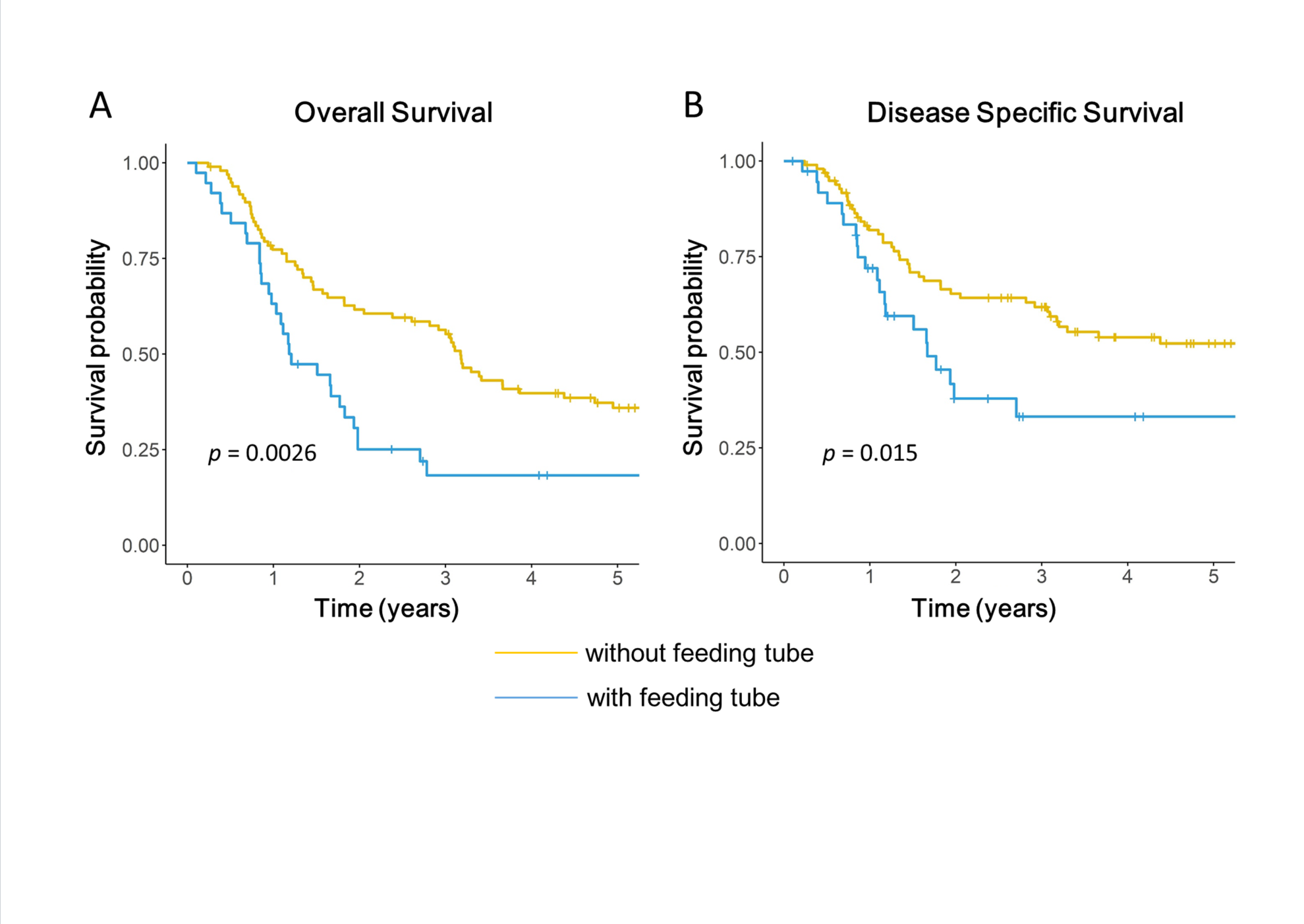

Supplement: Supplementary file 1 — Supplementary file1 (DOCX 732 kb) [file 10434_2022_12744_MOESM1_ESM.docx]
